# Complete Sequences of Six IncA/C Plasmids of Multidrug-Resistant Salmonella enterica subsp. enterica Serotype Newport

**DOI:** 10.1128/genomeA.00027-15

**Published:** 2015-02-26

**Authors:** Guojie Cao, Marc W. Allard, Maria Hoffmann, Steven R. Monday, Tim Muruvanda, Yan Luo, Justin Payne, Lydia Rump, Kevin Meng, Shaohua Zhao, Patrick F. McDermott, Eric W. Brown, Jianghong Meng

**Affiliations:** a Department of Nutrition & Food Science and Joint Institute for Food Safety & Applied Nutrition, University of Maryland, College Park, Maryland, USA; b Division of Microbiology, Office of Regular Science, Center for Food Safety & Applied Nutrition, U.S. Food & Drug Administration, College Park, Maryland, USA; c Biostatistics Branch, Center for Food Safety & Applied Nutrition, U.S. Food & Drug Administration, College Park, Maryland, USA; d Department of Microbiology & Immunology, Stanford School of Medicine, Stanford University, Palo Alto, California, USA; e Division of Animal & Food Microbiology, Office of Research, Center for Veterinary Medicine, U.S. Food & Drug Administration, Laurel, Maryland, USA

## Abstract

Multidrug-resistant (MDR) Salmonella enterica subsp. enterica serotype Newport has been a long-standing public health concern in the United States. We present the complete sequences of six IncA/C plasmids from animal-derived MDR *S*. Newport ranging from 80.1 to 158.5 kb. They shared a genetic backbone with *S*. Newport IncA/C plasmids pSN254 and pAM04528.

## GENOME ANNOUNCEMENT

Salmonella enterica subsp. enterica serotype Newport (*S*. Newport) is an important serotype that causes salmonellosis in humans and animals and has been implicated in several multistate outbreaks in the United States (1). *S*. Newport is differentiated into three lineages based on multilocus sequence typing (MLST) (2) and whole-genome sequencing data (1). The emergence and dissemination of multidrug-resistant (MDR) *S*. Newport has been a long-standing public health concern in the United States (3). Extended-spectrum cephalosporin (AmpC) MDR *S*. Newport strains harboring IncA/C plasmids showed a clonal structure in *S*. Newport lineage II (1).

Currently, there are two publicly available *S*. Newport IncA/C plasmid sequences, pSN254 (NC_009140) and pAM04528 (FJ621587) (3). However, little is known about the evolution, genetic diversity, and common characteristics of IncA/C plasmids of animal-derived MDR *S*. Newport. We selected six animal-derived MDR *S*. Newport strains: CVM22425 (cattle, Arizona), CVM22462 (canine, Arizona), CVM22513 (cattle, North Carolina), CVMN1543 (ground beef, Georgia), CVM21550 (swine, Texas), and CVM21538 (chicken, Georgia). We report the complete sequences of six IncA/C plasmids from these MDR *S*. Newport strains: pCVM22425 (158,195 bp), pCVM22462 (158,521 bp), pCVM22513 (120,346 bp), pCVMN1543 (118,585 bp), pCVM21550 (120,340 bp), and pCVM21538 (80,098 bp). These plasmids shared a genetic backbone with pSN254 and pAM04528.

*S*. Newport strains were cultured on Trypticase soy agar (TSA; Becton & Dickinson, NJ) and in Trypticase soy broth (TSB; Becton & Dickinson, NJ) overnight at 37°C. Genomic DNA was extracted using DNeasy blood and tissue kit (Qiagen, Valencia, CA). We used a Pacific Biosciences RSII (PacBio, Menlo Park, CA) system to obtain the complete sequences. The 10-kb libraries of each strain were sequenced using C2 chemistry kits on four single-molecule real-time (SMRT) cells with a 90-min collection protocol. Raw reads of sequencing data were assembled using the PacBio hierarchical genome assembly process 2 (HGAP2)/Quiver software package. In addition to chromosome sequences, complete sequences of four plasmids were obtained from the genomic DNA data assembly. They were denoted pCVM22425, pCVM22462, and pCVMN1543 from strains CVM22425, CVM22462, and CVMN1543, respectively.

DNA extraction of plasmids pCVM22513, pCVM21538, and pCVM21550 (from strains CVM22513, CVM21538, and CVM21550, respectively) was performed according to a procedure described by M. Hoffmann, S. Zhao, J. Pettengill, S. Ayers, J. Payne, J. Meng, M. Allard, P. McDermott, E. Brown, and S. Monday (unpublished data). Plasmids were initially transformed into Escherichia coli DH10Br and isolated using a Qiagen large-construct kit (Qiagen). The 10-kb insert libraries of each strain were sequenced using C2 chemistry kits on one SMRT cell with a 120-min collection protocol. Raw reads were assembled using the PacBio hierarchical genome assembly process 3 (HGAP3)/Quiver software package (4). These sequences were annotated using the NCBI Prokaryotic Genomes Annotation Pipeline (5).

### Nucleotide sequence accession numbers.

The complete plasmid sequences have been deposited in GenBank under the accession numbers CP009560 (pCVM22425), CP009567 (pCVM22462), CP009562 (pCVM22513), CP009563 (pCVM21538), CP009570 (pCVMN1543), and CP009564 (pCVM21550).
